# Pathogenesis of Common Ocular Diseases

**DOI:** 10.1155/2015/734527

**Published:** 2015-12-30

**Authors:** Jun Zhang, Jingsheng Tuo, Zhongfeng Wang, Aiqin Zhu, Anna Machalińska, Qin Long

**Affiliations:** ^1^Synaptic Physiology Section, National Institute of Neurological Disorders and Stroke, National Institutes of Health, Bethesda, MD, USA; ^2^Laboratory of Immunology, National Eye Institute, National Institutes of Health, Bethesda, MD, USA; ^3^Institutes of Brain Science, State Key Laboratory of Medical Neurobiology, Collaborative Innovation Center for Brain Science, Fudan University, Shanghai, China; ^4^Institute of Geriatrics, Qinghai Provincial Hospital, Xining, Qinghai, China; ^5^Department of Ophthalmology and Department of Histology and Embryology, Pomeranian Medical University, Szczecin, Poland; ^6^Department of Ophthalmology, Peking Union Medical College Hospital, Chinese Academy of Medical Sciences & Peking Union Medical College, Beijing, China

The most common diseases resulting in irreversible blindness or vision impairment include age-related macular degeneration (AMD), glaucoma, diabetic retinopathy, cataract, and dry eye. These diseases seriously affect the quality of life in elderly people worldwide. Therefore, understanding their pathogenesis and the development of strategies allowing earlier detection and treatment demands more effort and attention from both basic and clinical fields. This issue focuses on different aspects of these diseases in elderly population.

AMD is one of the most common diseases resulting in irreversible blindness worldwide in the elderly population. Among multifactorial pathogenesis, immune dysregulations are leading theories of AMD pathogenesis. Recently IL-17 pathway was reported to be involved in AMD pathogenesis. In the original article “Responses of Multipotent Retinal Stem Cells to IL-1*β*, IL-18, or IL-17” by S. Chen et al., the authors investigated the responses of multipotent retinal stem cells (RSCs), isolated from mice, to the proinflammatory signaling molecules including IL-1*β*, IL-18, and IL-17A. They found that the addition of IL-1*β*, IL-18, or IL-17A in the cultured cell decreased RSC viability but increased pyroptotic and/or necroptotic cells. The study is innovative and unique, because, instead of RPE19, a new cell type was used as the model system. Additionally, the results fill gaps in understanding immunological mechanism of AMD pathogenesis.

Like AMD, glaucoma and diabetic retinopathy are common causes of blindness in older adults. Glaucoma is often caused by damage to the optic nerve due to an abnormally high pressure in your eye, while diabetic retinopathy is a diabetes complication, caused by damage to the retinal blood vessels. However, both of them have no symptoms or warning signs at early stage. Thus, it is important to have regular eye exams to measure intraocular pressure and ocular blood flow. In the review paper “Ocular Blood Flow Autoregulation Mechanisms and Methods” by X. Luo et al., the authors summarized the methods for ocular blood flow evaluating and discussed mechanism and treatment of ocular blood flow regulation, particular in glaucoma and diabetic retinopathy.

Cataract is one of the major causes of visual impairment of elderly people. Although recent bioinformatics studies revealed susceptibility genes, such as EPHA2, for age-related cataract, the mechanism underlying its pathogenesis remains elusive. In the original paper “The Polymorphisms with Cataract Susceptibility Impair the EPHA2 Receptor Stability and Its Cytoprotective Function” by J. Yang et al., the authors found that EPHA2 signaling can protect the lens epithelial cells from oxidative stress-induced cell death. In the original paper “Epigenetic Regulation of Werner Syndrome Gene in Age-Related Cataract” by X. Zhu et al., the authors investigated the promotor methylation and histone medication of Werner syndrome gene (WRN). They found that both mRNA and protein levels of WRN were significantly decreased only in anterior lens capsules in age-related cataract, suggesting that the strategies to intervene epigenetic alteration in this disease should aim to anterior lens capsules. By investigating very large cataract patient population in rural China, X. Cao et al. presented a normative ocular biometry of adult cataract patients in rural China. They found that the axial length is normally distributed with a positive skew and a big kurtosis and corneal astigmatism may affect rural Chinese vision acuity.

Dry eye is a multifactorial disorder of the tears and ocular surface and is a common and often unrecognized disease affecting tens of millions of individuals worldwide. Q. Long et al. evaluated the biomechanical behavior of the cornea in dry eye using, for the first time, Corneal Visualization Scheimpflug Technology (CorVis ST), a new noncontact tonometry system. Their results provide insight into its full usefulness for dry eye patients. B. Wang et al. compared dry eye disease that resulted from two refractive surgeries [small-incision lenticule extraction (SMILE) versus femtosecond laser in situ keratomileusis (FS-LASIK)] in high myopia. They found that SMILE is a safe and successful alternative for the correction of refractive error and may provide a more superior and safer refractive outcome than FS-LASIK in the first six months following surgery. CorVis ST, the very latest technology, has been used by J. Wang et al. to assess the biomechanical parameters of the cornea in myopic and emmetropic eyes.

Vitreous hemorrhage (VH) is one of the ophthalmologic emergency situations. In the paper contributed by D. Y. Kim and colleagues, the authors analyzed causes and prognosis of acute-onset preoperatively unknown origin VH in 169 eyes and found that retinal vein occlusion, retinal break, and AMD are the most common causes. In addition, aging may be an important factor for influencing visual prognosis following vitrectomy.

Optic neuritis is one of the common optic neuropathies and is highly associated with multiple sclerosis. In the original paper “Evaluation of Retinal Nerve Fiber Layer and Ganglion Cell Complex in Patients with Optic Neuritis or Neuromyelitis Optica Spectrum Disorders Using Optical Coherence Tomography in a Chinese Cohort” by G. Tian et al., the authors reported that spectral-domain optical coherence tomography, SD-OCT, is a very useful and objective method to evaluate the thickness of the peripapillary retinal nerve fiber layer and macular ganglion cell complex in optic neuritis and neuromyelitis optica.

Common age-related ocular diseases demand attention as a global health problem. This special issue covered pathogenesis, diagnosis, and treatment of most of these diseases.

*Jun Zhang* *Jun Zhang* *Jingsheng Tuo* *Jingsheng Tuo* *Zhongfeng Wang* *Zhongfeng Wang* *Aiqin Zhu* *Aiqin Zhu* *Anna Machalińska* *Anna Machalińska* *Qin Long* *Qin Long*

